# Influence of acidic metabolic environment on differentiation of stem cell-derived cardiomyocytes

**DOI:** 10.3389/fcvm.2024.1288710

**Published:** 2024-03-20

**Authors:** Mao Jiahao, Zhou Fan, Mu Junsheng

**Affiliations:** ^1^ Department of Cardiac Surgery, Beijing Institute of Heart Lung and Blood Vessel Diseases, Beijing Anzhen Hospital, Capital Medical University, Beijing, China; ^2^ Department of Ultrasound, The Third Medical Center of PLA General Hospital, Beijing, China

**Keywords:** pH value, lactate, stem cells, cardiac differentiation, acidic metabolic environment

## Abstract

Stem cell-based myocardial regeneration is a frontier topic in the treatment of myocardial infarction. Manipulating the metabolic microenvironment of stem cells can influence their differentiation into cardiomyocytes, which have promising clinical applications. pH is an important indicator of the metabolic environment during cardiomyocyte development. And lactate, as one of the main acidic metabolites, is a major regulator of the acidic metabolic environment during early cardiomyocyte development. Here, we summarize the progress of research into the influence of pH value and lactate on cardiomyocyte survival and differentiation, as well as related mechanisms.

## 1 Introduction

Myocardial infarction is a common and often fatal heart disease. Most myocardial infarction occurs due to a dramatic reduction or interruption of coronary blood supply because of coronary lesions, leading to persistent ischemia and consequent necrosis of cardiomyocytes. In the past, myocardial infarction was more common in developed areas such as Europe and the United States. However, the incidence of myocardial infarction has gradually increased in low- and middle-income countries over the past three to four decades (1). Due to the poor regenerative capacity of cardiomyocytes, massive necrosis of these cells leads to cardiac fibrosis and heart failure (2). Current treatments for heart failure include pharmacological therapy, cardiac resynchronization therapy, left ventricular assistant device, and cardiac transplantation. However, modern drug therapy and assistant device therapy cannot reverse the necrosis of cardiomyocytes and only inhibit or delay disease progression. Accordingly, in the past few decades, myocardial regeneration has become a cutting-edge topic in the treatment of myocardial infarction and heart failure, mainly including *in vitro* cultured cardiomyocyte transplantation, cardiac tissue engineering, stimulation of endogenous cardiomyocyte proliferation, and reprogramming of non-myocytes into cardiomyocytes (2).

**Figure 1:**
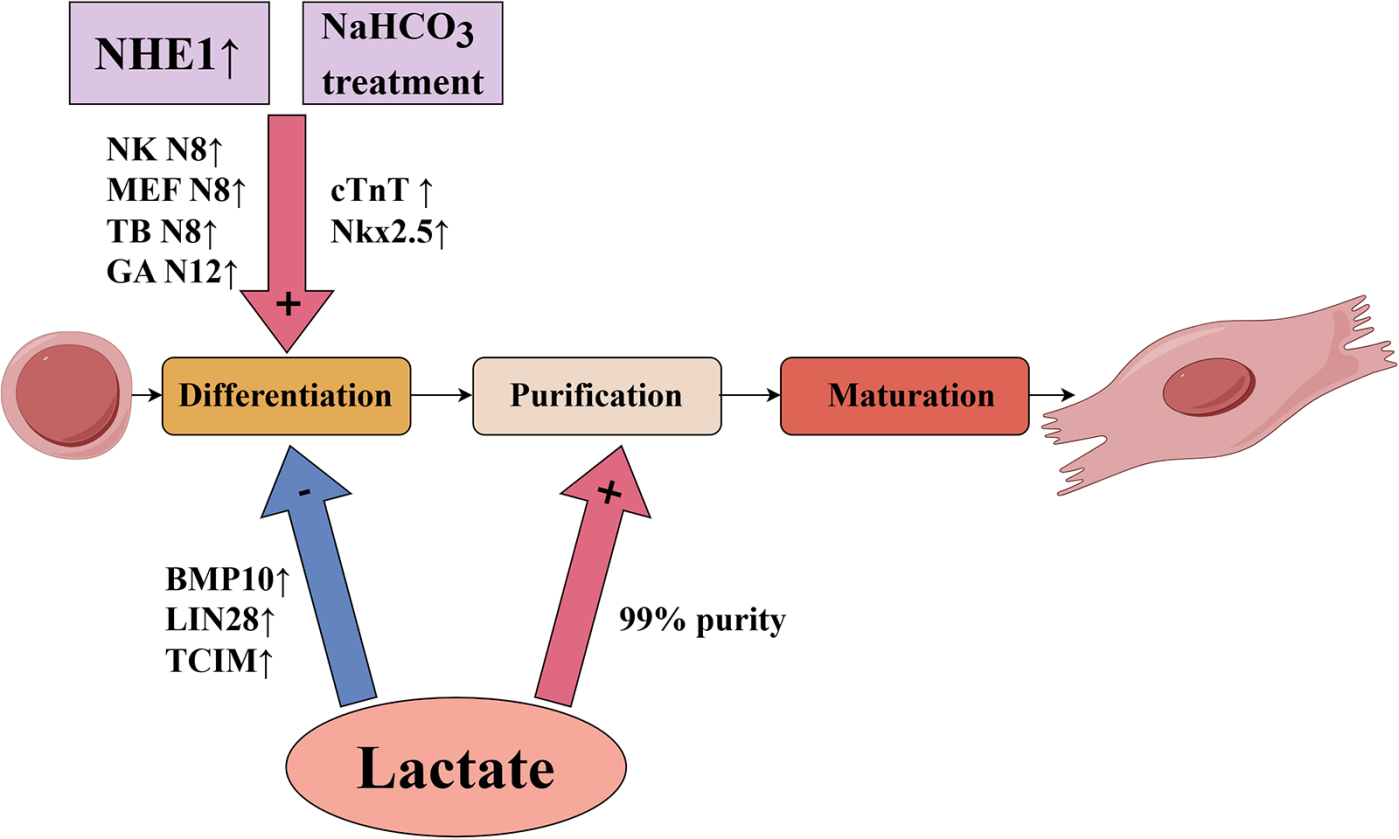
Influence of pH value and lactate on key technologies of *in vitro* culture of pluripotent stem cell-derived cardiomyocytes.

For *in vitro* culture of cardiomyocytes, pluripotent stem cells, including embryonic stem cells and induced pluripotent stem cells, are a potential source for generating cardiomyocytes for therapeutic use (3).There are several critical technologies that need to be investigated and advanced from the *in vitro* cultivation of cardiomyocytes through the injection of cardiomyocytes into the infarcted area of the heart. The primary challenge of *in vitro* cultured cardiomyocyte transplantation is overcoming the ischemic environment that can cause transplantation failure. Additionally, approximate 10^9^ cardiomyocytes per person would be needed for heart regenerative therapies, so the following technical considerations would be necessary: yield expansion of genetically and epigenetically stable pluripotent stem cells, effective and reproducible differentiation, and highly reliable cell purification and maturation (4). Alternatively, cardiac *in situ* tissue engineering uses methods such as recombinant virus, interfering RNAs, or modulation of the cellular metabolic environment to stimulate proliferation of endogenous cardiomyocytes (2, 5). However, the mammalian heart has a transient capacity for self-repair that occurs only during the neonatal period (6, 7). The renewal rate of cardiomyocytes in adult mammals is very low and insufficient for regeneration after myocardial infarction (8, 9), especially because existing cardiomyocytes are the likely source of newly generated cardiomyocytes (9). An emerging trend in myocardial regeneration strategies is manipulating the metabolic environment to induce activation of stem cells. Many studies have demonstrated that the cellular metabolic environment influences cellular behavior and has an important role in the differentiation process of cardiomyocytes (10, 11). In this review, we focused on the effects of pH value and lactate on cardiomyocyte survival and differentiation, which are briefly summarized in Figure 1 and Table 1.

**Table 1:** Recent studies focused on the effects of acidic metabolic environment on cardiomyocyte survival and differentiation.

Publication year & main author	Cardiomyocyte types	Indicator of acidic metabolic environment	Effects on survival and differentiation	Mechanis-ms
2007 Cohen (12)	Cardiomyocytes of myocardial infarction in isolated rabbit hearts	Low pH value	Reduction of myocardial infarction zone	Inhibition of MPTP formation
2009 Li (13)	Embryonic stem cell-derived cardiomyocytes	NHE1 activity	Promotion of cardiomyocyte differentiation	–
2018 Liu (14)	Human pluripotent stem cell-derived cardiomyocytes	Suppression of medium acidosis	Promotion of cardiomyocyte differentiation	–
2022 Ordono (5)	Mouse neonatal cardiomyocytes; human induced pluripotent stem cell-derived cardiomyocytes	Lactate	Promotion of cardiomyocyte dedifferentiation and proliferation	–
2022 Du (15)	Neonatal rat ventricular myocytes	Lactate	Promotion of cardiomyocyte proliferation; Swtich from oxidative phosphorylation to glycolysis	Lactate-La-cRS2 signaling pathway
2013 Tohyama (16)	Mouse and human pluripotent stem cell-derived cardiomyocytes	Lactate	Promotion of cardiomyocyte purification and proliferation	–

## 2 PH and survival of cardiomyocytes

### 2.1 Mechanisms of ischemic injury and protection of low pH value

Due to the inflammation caused by acute myocardial infarction, the local pH of the infarcted zone is often lower than the physiological pH of normal cardiac tissue (12, 17). Ischemia leads to tissue hypoxia, resulting in acidosis from anaerobic glycolysis of cells; in contrast, reperfusion reoxygenates the myocardial infarcted zone and promotes the recovery of physiological pH (17). Although acidosis is often considered detrimental to cell survival, numerous studies have shown that acidosis protects against ischemic and hypoxic injury in cardiomyocytes, hepatocytes, and other cells; conversely, reperfusion following prolonged ischemia may cause cell death, an incongruity known as the “pH paradox” (18, 19).

Oxidative stress occurs in hypoxic and ischemic tissues after reperfusion and is accompanied by a decrease of reduced glutathione, increase in oxygen radical species, and increase in cytoplasmic Ca^2+^ (20). When cells are pathologically loaded with high levels of Ca^2+^, permeability of the intracellular mitochondrial membrane increases nonspecifically (20, 21), resulting in the swelling and uncoupling of mitochondria. This process, known as mitochondrial permeability transition, leads to the formation of mitochondrial membrane permeability transition pores (MPTP). Isolated heart cells reoxygenated after long-term hypoxia undergo irreversible cell death and necrosis (20) due to the activation of Ca^2+^-dependent proteases, phospholipases, and nucleases. Mitochondria with MPTP have difficulty producing ATP to renovate the damage caused by these enzymes. Moreover, these swollen mitochondria hydrolyze the ATP produced by glycolysis and residual functional mitochondria (21). There is ample evidence that a low intracellular pH preserves cells from irreversible damage during post-ischemic reperfusion (20) and many studies suggest that this protection occurs through inhibition of MPTP formation (12, 21, 22). Cohen et al. (12) conducted an experiment on isolated rabbit hearts with 30 min of local ischemia and found that compared with direct reperfusion, use of acidic perfusion solution 2 min before reperfusion significantly reduced the size of myocardial infarction, yielding a protective effect on the heart.

### 2.2 Low pH value and stem cell-derived cardiomyocyte transplantation

The development and design of *in vitro* cultured cardiac tissue must consider the specific environment of myocardial ischemia, otherwise cardiomyocytes cultured in a highly controlled environment are likely to undergo necrosis when implanted *in vivo* (5). Long-term transplantation is not possible when stem cells are directly injected into the infarcted area due to low cell retention (23, 24), usually caused by the local ischemic environment and lack of anchoring conditions for cell survival (25). To address this issue, Li et al. (25) developed a pH- and temperature-sensitive hydrogel as a delivery vehicle for *in vitro* cultured cardiomyocytes that could solidify at the pH of the infarcted zone but could not solidfy at the pH of blood. Their research also found that the hydrogel could ensure cell retention and cardiomyocyte differentiation was significantly impacted by the hydrogel. Cardiomyocytes in the hydrogel exhibited increased expression of cTnT, MYH6, and CACNA1c, compared to those in a 2D culture plate. In conclusion, low pH due to myocardial infarction provides guidance for *in vitro* cultured cardiomyocyte transplantation.

## 3 PH and differentiation of stem cell-derived cardiomyocytes

### 3.1 Intracellular pH and differentiation of stem cell-derived cardiomyocytes

Cardiomyocyte function depends on the intracellular pH, which is altered under certain physiological and pathological circumstances. These changes require compensatory mechanisms to return the intracellular pH to its resting level (7.1–7.4) (26). Two main ion transport proteins are activated when intracellular pH decreases in cardiomyocytes: the Na^+^/H^+^ exchanger (NHE) and Na^+^-HCO_3_^−^ co-transporter (26). NHE is a common plasma membrane glycoprotein that regulates intracellular pH by expelling an intracellular proton in exchange for an extracellular sodium ion, thereby removing excessive intracellular acid (13). Although there are nine isoforms of NHE, NHE1 is the only plasma membrane isoform in the heart (27). An assessment of the contribution of each transporter protein to removing intracellular acid indicated that NHE1 is the major transporter protein reducing intracellular acid loads in cardiomyocytes (28). Additionally, Na^+^-HCO_3_^−^ co-transporter proteins were found to be more active when the intracellular pH is >7, suggesting that NHE1 is a more critical pH regulator protein when intracellular pH is <7 in cardiomyocytes (29).

Notably, expression of NHE1 protein increases during cardiac development and for a very short period after birth (30). When cell differentiation declines after birth, NHE1 activity decreases (30, 31). Collectively, these findings suggest that NHE1 may play an important role in the development and differentiation of cardiomyocytes.

A significant source used for cardiac tissue transplantation is embryonic stem cells, which have great potential for use in regenerative therapies because they can differentiate into virtually all cell types. Li et al. (13) first studied the effects of NHE1 on embryonic stem cell differentiation by using the mouse stem cell line CGR8 to form embryoid bodies (EBs) and cardiomyocytes. From day 8 to day 12, cells were treated with the NHE1 specific inhibitor EMD887580, which significantly decreased the percentage of beating EBs compared with the control group. In addition, NHE1 inhibition by EMD887580 caused a significant decline of α-myosin heavy chain protein expression. More importantly, the transcription factors Nkx2.5 and Tbx5, two markers of myocardial differentiation (32), were significantly decreased at day 12 of treatment with EMD887580. These results suggest that inhibition of NHE1 activity prevents the differentiation of embryonic stem cells into cardiomyocytes. Furthermore, Li et al. (13) infected CGR8 cells with an adenovirus containing NHE1 to enhance NHE activity. Compared with the control group, infection increased the percentage of beating EBs and expression levels of several cardiomyocyte marker transcription factors, such as NK N8, MEF N8, TB N8 and GA N12. Altogether, these results suggest that NHE1 activity is important for promoting differentiation of stem cells into cardiomyocytes.

Notably, the NHE1 specific inhibitor EMD887580 did not affect NHE1 expression levels and only inhibited NHE1 activity and the myocardial differentiation process, suggesting that changes in NHE1 expression levels may be independent of the myocardial differentiation process and NHE1 is not a concomitant product of cardiomyocyte differentiation (13). These results further emphasize the promoting effect of NHE1 on differentiation of stem cells into cardiomyocytes. However, the mechanism by which this effect occurs is unclear and further studies are needed to investigate the trigger mechanisms and signaling pathways by which NHE1 activity affects cardiomyocyte differentiation.

### 3.2 Extracellular pH and differentiation of stem cell-derived cardiomyocytes

In the actual culture of human pluripotent stem cells for differentiation into cardiomyocytes, cell culture is often performed at higher cell densities in order to obtain a certain cell yield. However, high-density cell culture will unavoidably result in the buildup of lactate, particularly in some differentiation protocols that use differentiation medium containing glucose. In these cases, the lactate produced by the cells through glycolysis will cause a certain level of acidosis, which will ultimately result in cell damage and death. Liu et al. (14) reported that low medium pH caused by high-density culture of human pluripotent stem cells inhibited glucose consumption and led to cell cycle arrest followed by cell death, whereas supplementation of sodium bicarbonate (NaHCO_3_) inhibited acidosis and improved cell survival.NaHCO_3_ treatment significantly promoted the differentiation of pluripotent stem cells to cardiomyocytes, which was supported by elevation of cardiomyocyte markers cTnT and Nkx2.5.This suggests that the addition of NaHCO3 can increase buffer capacity and improve cardiomyocyte differentiation in high-density culture, which is important for actually obtaining a certain yield of *in vitro* cultured cardiomyocytes.

### 3.3 Summary

In conclusion, both the reduction of intracellular acid load in cardiomyocytes by NHE1 and the inhibition of acidosis in the extracellular environment of human pluripotent stem cell-derived cardiomyocytes by the addition of NaHCO_3_ promote the differentiation of cardiomyocytes.These results suggest that cardiomyocyte differentiation is associated with the improvement of acidic metabolic environment.

## 4 Lactate and differentiation of stem cell-derived cardiomyocytes

### 4.1 The role of lactate in early cardiomyocyte development

The mammalian heart has an extreme energy requirement because it requires constant contraction to maintain the oxygen supply to all organs of the body. When the mammalian heart has numerous substrates available, it can use all of them to produce energy, including glucose, ketones, amino acids, and lactate (33). During early pregnancy, the human embryo and placenta develop in a hypoxic environment (34–36). The placenta produces large amounts of lactic acid, which becomes an important substrate for early fetal development (36). The fetal heart depends heavily on glycolysis during early development and primarily uses lactate as an energy source (37). Lactate transport through the plasma membrane is essential for early heart cell metabolism and pH regulation. Monocarboxylate transporter protein (MCT) oversees lactate transport, scavenging lactate produced by glycolysis and allowing cells capable of utilizing lactate for uptake, such as cardiomyocytes (34). Upon analyzing MCT-1 and MCT-4 expression in the developing chicken embryo heart, Han et al. (34) found that MCT-4 was expressed throughout the early embryonic and fetal stages, indicating that cardiac precursor cells can use lactate for glycolysis in a hypoxic environment.

Compared with the adult heart, fetal cardiomyocytes have a greater proliferative capacity and less-differentiated phenotype. After birth, cardiomyocytes in an aerobic environment stop proliferating and begin to become hypertrophic (5). In addition, a large amount of oxygen enters the heart, which changes the metabolic environment of cardiomyocytes. As a result, cellular glycolysis and lactate levels decrease, the mitochondrial oxidation capacity of cardiomyocytes increases, and fatty acid β-oxidation becomes the main energy source of the heart after birth (38). Cardiomyocytes enter the terminal stage of differentiation along with the metabolic transition from glycolysis to oxidative phosphorylation (39), and eventually acquire a mature differentiated state. In summary, the differentiation process of cardiomyocytes primarily occurs in a uniquely hypoxic and lactic acid-rich metabolic environment.

### 4.2 The effects of lactate on differentiation of stem cell-derived cardiomyocyte

Many recent studies have shown an important role for lactate in the differentiation process of cardiomyocytes. Ordono et al. (5) studied the effects of lactate on neonatal cardiomyocytes and cardiomyocytes derived from human induced pluripotent stem cells. Assessments of the expression of Ki67 (a marker of cell proliferation) and Aurora-B kinase (a key regulator of cytokinesis during mitosis) indicated that both sources of cardiomyocytes had significantly increased Ki67+cTnT+ and AurB+cTnT+ cells in response to lactate. These results suggest that the presence of lactate can increase cardiomyocyte cycle activity and stimulate their proliferative activity. In addition, lactate had no effect on the number of fibroblasts, suggesting that the proliferation-inducing effect of lactate is specific to cardiomyocytes and does not affect other cardiac cell populations. The results of RNA sequencing and gene expression analysis indicate that cardiomyocytes cultured with lactate upregulated expression of bone morphogenic protein 10 (BMP10), LIN28, and TCIM; and downregulated expression of GRIK1 and DGKK. Bmp10 is a secreted protein that promotes cardiomyocyte proliferation and inhibits cardiac cell maturation (40, 41). Lin28 is associated with stem cell metabolism and pluripotency (10) and regulates the activation of early cardiomyocytes (42), while TCIM is associated with a poor differentiation state of cells (43). Grik1 is thought to restrain cell proliferation (44), while Dgkk is associated with cardiomyocyte hypertrophy (45). These results further support a role for lactate in stimulating cardiomyocyte proliferation at the gene expression level. To understand the mechanisms of lactate regulation on cardiomyocytes, their team used the human gene database GeneCards® to match genes that were upregulated or downregulated in human induced pluripotent stem cell-derived cardiomyocytes. They found that most transcription factor binding sites associated with these genes were related to hypoxia signaling pathways; among these, SP1 (46), MEF2A (47), and CREM (48) have been implicated in the protection or proliferation of cardiomyocytes under hypoxic conditions. These results suggest that the effect of lactate may be related to the activation of hypoxia signaling pathways in cardiomyocytes. In summary, the results described above suggest that lactate keeps cardiomyocytes in a dedifferentiated stage to promote their proliferative activity through gene-level regulatory mechanisms.

Du et al. (15) reported a chemical mixture of five small molecules that effectively enabled cardiomyocytes to re-enter the cell cycle and undergo cytokinesis, thereby inducing cardiomyocytes to de-differentiate before entering proliferation. Notably, this chemical mixture directly raised lactate levels in the culture medium and increased numbers of cardiomyocytes entering the cell cycle, where the intracellular lactate concentration nearly doubled. Analysis of changes in metabolic gene expression indicated an increase in the level of glycolysis and decrease in the level of oxidative phosphorylation in cardiomyocytes. Du et al. (15) further showed that lactate can inhibit two mitochondrial proteins through LacRS2-mediated lactylation, including pyruvate dehydrogenase α 1 (*PDHA1*) and carnitine palmitoyltransferase 2 (*CPT2*), two key enzymes catalyzing the production of acetyl coenzyme A from pyruvate and oxidation of long-chain fatty acids, respectively. Interestingly, the chemical mixture described above could increase lactylation of both PDHA1 and CPT2. Furthermore, knockdown of LacRS2 siRNA or inhibition of LacRS2 with β-alanine reduced the level of lactylated PDHA1 and number of cardiomyocytes entering the cell cycle. These results suggest that the above-described chemical mixture likely promoted dedifferentiation and proliferation of cardiomyocytes through the lactate-LacRS2 downstream signaling pathway, which induces cardiomyocyte proliferation and allows a shift in cellular metabolism from oxidative phosphorylation to glycolysis.

Pluripotent stem cells include embryonic stem cells and induced pluripotent stem cells, both of which are potential sources for generating cardiomyocytes for therapeutic use (3). When embryonic stem cells are cultured *in vitro*, differentiated cardiomyocytes usually constitute only a small fraction of the cells present in EBs of embryonic stem cells (49). Tohyama et al. (16) used glucose-free medium rich in lactate to culture derivatives of pluripotent stem cells and found that only cardiomyocytes survived, yielding cultures with 99% purity. Gene expression analysis indicated elevated mRNA expression of the cardiomyocyte-associated gene *MYH6*, complete elimination of mRNAs for non-cardiac genes (*NANOG*, *MYOD*, *AFP*, and *MAP2*), and significant enrichment of mRNAs for other cardiomyocyte-associated genes (*ACTC1* and *NKX2.5*).In addition, they found that cardiomyocytes purified with lactate proliferated up to 2.5-fold within 8 days of cell culture, and the proportion of multinuclear cardiomyocytes increased over time compared to day 2. These results suggest that pluripotent stem cell-derived cardiomyocytes purified with lactate show enhanced proliferation.

### 4.3 Non-consistent relationship between lactate and pH value

It's important to note that Tohyama's team selected 4 mM after optimizing the lactate concentration since it produced the highest cardiomyocyte purity and yield on days 20 to 30 of differentiation and had the lowest number of dead cells in cell culture. Additionally, lactate supplementation has the potential to lead to acidification of the intracellular as well as extracellular environment in the medium. They investigated whether 4 mM lactate affects pH, and their experiments confirmed that the extracellular pH stabilized at 7.5 after 1 h of incubation in a 5% CO2 incubator and that the intracellular pH of the cardiomyocytes was unaffected by the addition of 4 mM lactate. These results suggest that the effect of lactate on pluripotent stem cell-derived cardiomyocytes is not pH-dependent but is more likely a consequence of lactate itself.

### 4.4 Summary

In conclusion, lactate, as the main energy substrate for early cardiac development, enables cardiomyocytes to acquire a dedifferentiated phenotype and enhances their proliferative capacity through regulation at the gene level. There are broad therapeutic prospects for cardiac *in situ* tissue engineering (e.g., stimulating the proliferation of endogenous cardiomyocytes through lactate), which provides a new possibility for myocardial regeneration. Moreover, for *in vitro* culture of cardiomyocytes, lactate can effectively improve both the proportion and yield of cardiomyocytes differentiated from stem cells. Compared with other induction methods, use of lactate is simpler, more effective, and has great significance for practical applications of cardiomyocyte culture.

## 5 Perspectives and future outlook

Acidic metabolic environment has an important role in the differentiation of cardiomyocytes. The intracellular pH of cardiomyocytes is mainly regulated by NHE1, whose enhanced activity promotes cardiomyocyte differentiation, although the molecular mechanism of this process has not been elucidated. For *in vitro* culture of cardiomyocytes, inhibition of high-density culture-induced acidosis improves cardiomyocyte differentiation, while the related mechanism still needs to be explored. In the fetal heart, the metabolic environment during early development of cardiomyocytes is rich in lactate, which mainly determines the acidic metabolic environment. Lactate can promote the dedifferentiation of cardiomyocytes and enhance their proliferation ability. For *in vitro* culture, lactate can effectively increase both the proportion and yield of cardiomyocytes differentiated from stem cells. Regulation of the cellular metabolic environment by lactate has broad therapeutic prospects in practical clinical applications, however the specific mechanisms of related effects remain to be investigated.

It is worth noting that in order to realize the clinical application of the topic of myocardial regeneration, stability and safety-related technical issues need to be thoroughly verified.Recently, Halloin et al. (50, 51) reported stirred tank bioreactor (STBR) technology. It can achieve yield expansion of human pluripotent stem cells with pluripotency and karyotype stability and effective cardiomyogenic differentiation by merging metabolic control of pH value and lactate level.

The majority of the aforementioned studies focus on the effects of pH value and lactate on the early stage of stem cell-derived cardiomyocyte differentiation.For the further maturation of stem cell-derived cardiomyocytes, the role of pH value and lactate has not been thoroughly investigated.The influence of pH value and lactate culture on maturation of stem cell-derived cardiomyocytes at the levels of cell morphology, electrophysiology, contractile function, and metabolic function may be a new research direction in the field.

## 6 Conclusions

Acidic metabolic environment has important influence on the survival and differentiation of cardiomyocytes. PH value is an important indicator of acidic metabolic environment. Both reduction of intracellular acid load and inhibition of extracellular acidosis can improve cardiac differentiation. Lactate, as the main regulator of metabolic environment during early cardiomyocyte development, induces dedifferentiation and proliferation of cardiomyocytes.
